# Atypical Neuropsychiatric Presentation in a Patient Expecting Liver Transplantation

**DOI:** 10.1155/2018/4609631

**Published:** 2018-07-09

**Authors:** Laury Chamelian, Austin Pereira, Kiran Grant, Catherine Maurice

**Affiliations:** ^1^Department of Psychiatry, Division of Neuro-Psychiatry, Centre Hospitalier de l'Université de Montréal, Canada; ^2^Faculty of Medicine, University of Toronto, Canada; ^3^Department of Medicine, Division of Neurology, University Health Network, Pencer Brain Tumour Centre, Princess Margaret Hospital, Canada

## Abstract

Patients presenting with acute or chronic hepatopathy can develop altered mental status with psychomotor slowing, most commonly indicating encephalopathy. We present the case of a 56-year-old patient who developed subacute atypical neuropsychiatric symptoms including cognitive and behavioural disorganization, manic-like state, and lateralized parkinsonian syndrome. The sequence of events, complete work-up, and detailed neuropsychiatric examination were not compatible with hepatic encephalopathy or delirium; therefore we extended our differential diagnosis and suggested the pathophysiological process described below.

## 1. Case Report

A 56-year-old female was diagnosed with primary biliary cirrhosis after presenting with pruritus and fatigue. Prior to this diagnosis, she worked as a business executive functioning at a high cognitive baseline. Jaundice and refractory ascites developed in the month prior to admission. Cognitive decline evolving over one month mandated her to take leave from work. She was referred to a tertiary centre specializing with hepatobiliary expertise. Her past medical history included arterial hypertension and gastroesophageal reflux, as well as cervicogenic headaches. She did not have psychiatric, legal, or relevant family history. Her baseline included diuretics, lactulose 10 ml TID, sodium benzoate 3g OD, metronidazole 250 mg BID, calcium carbonate 500 mg BID, and ursodiol 500 mg BID. Risperidone 1 mg OD and quetiapine 50 mg HS were prescribed at the time of referral but introduced after the neuropsychiatric presentation. No correlation could be established with her behavioural change and the pharmacotherapy after a meticulous review.

A general work-up was completed at the time of admission including albumin 34 g/L (N= 37-48 g/L), ammonia 20 mcg/dl (N= 15-45 mcg/dl), GGT 331 UI/L (N= 7-55 UI/L), ALT 74 U/L (N=9-30 U/L), AST 74 U/L (N=13-39 U/L), alkaline phosphatase 565 UI/L (N=36-110 UI/L), and INR 1.1. Psychiatry and neurology consultants reached the same conclusion: the patient's neuropsychiatric symptoms were atypical for hepatic encephalopathy [1]. She presented with personality alteration, psychomotor agitation, elevated mood, incongruous affect, ideoaffective discordance, and tangential, noninformative, and logorrheic speech as well as slightly decreased judgement. However, her orientation and insight were surprisingly intact. She scored 17/30 on the MOCA signifying a global cortical impairment. Disorganization and perseveration, along with auditory and somesthetic hallucinations, improved with neuroleptics. To provide an example, when this patient was informed of an upcoming neurology assessment, she completed her* “homework”* consisting of drawings and symbols. The neurological examination was unremarkable, mentioning absence of ophthalmoplegia, nystagmus, areflexia, gait instability, or primitive reflexes. The possibility of a diffuse cortical cognitive alteration with repercussion on expression, speech, memory, and personality was considered. Latent schizophrenia was clearly excluded, which would have been a contraindication to liver transplantation. However, a manic episode due to an organic disease was investigated.

**Figure 1:**
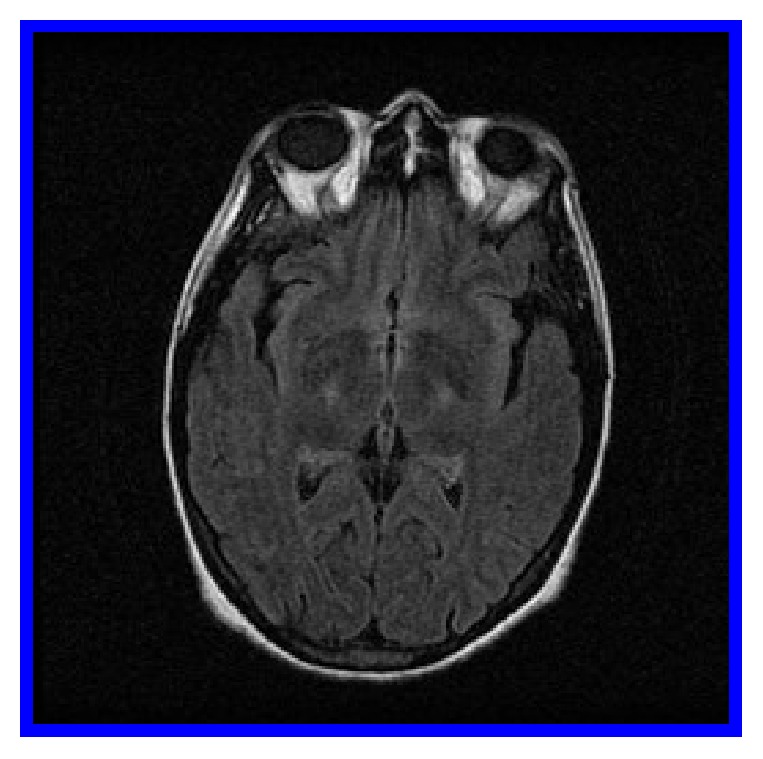
Axial brain MRI, FLAIR sequence, showing bilateral hyperintensities of the basal ganglia prior to transplantation.

**Figure 2:**
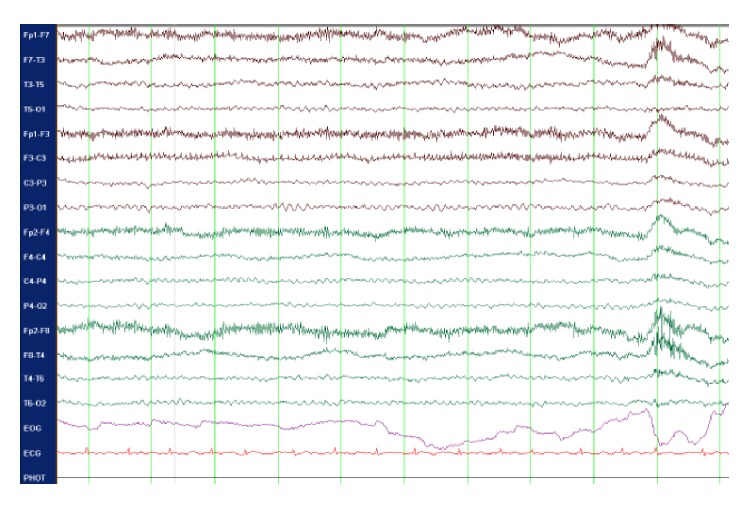
EEG showing global slow cerebral activity without epileptiform features.

Over the following weeks, she remained significantly disorganized, despite the fact that PRN doses of olanzapine and haloperidol managed her agitation. Lithium and regular doses of olanzapine were introduced, without success. An extensive work-up was performed, confirming Child-Pugh class C liver cirrhosis through biopsy. A brain MRI featured bilateral basal ganglia hyperintensities (Figure 1). Several EEGs were performed within her two-month hospitalization. The initial two EEG studies were unremarkable, without triphasic waves, slowing of the baseline rhythm, or epileptiform features. This would be unusual for hepatic encephalopathy, considering the extent of the neuropsychiatric symptoms; hepatic encephalopathy would at least have been classified as grade 2 [2]. However pathognomonic signs encountered at earlier stages were absent: shortened attention span, lethargy, dyspraxia, altered sleep rhythm, irritability, dysarthria, and asterixis. Repeated EEGs performed two and three months after onset of neuropsychiatric symptoms, respectively, showed a discrete intermittent global slow cerebral dysfunction (Figure 2). Cerebral SPECT and PET scans were essentially normal for patient age, showing no signs of significant atrophy. CSF analysis was essentially unremarkable. A thorough search for an underlying neoplasm included a thoracic CT scan, which demonstrated a calcified nodule at the right apex (PPD negative). It also included an abdo-pelvic CT scan, ascites analysis/culture, and alpha-fetoprotein level, all of which were negative. CSF, urine, ascites, and blood cultures were consistently negative. The inflammatory and autoimmune work-up was positive for ANA (1/640) and antimitochondrial antibodies (1/320). Otherwise, negative results were obtained for ENA, parietal cells antibodies, smooth muscle antibodies, TSH, thyroglobulin antibodies, transglutaminase antibodies, and protein electrophoresis. Copper intoxication was ruled out, as well as several indolent infections: HIV serology, anti-HCV, anti-HVA, anti-HBs, HBs-Ag, anti-HBc, Cryptococcus, VZV, and HSV.

She was discharged five months after her initial admission, preparing for a liver transplantation two months later. The surgery was uneventful. Interestingly, her husband noted a new tremor involving the left lower extremity. An asymmetrical parkinsonian akineto-rigid syndrome appeared three weeks prior to the transplantation. This extrapyramidal syndrome occurred before the introduction of immunosuppressive agents (tacrolimus, azathioprine, and methylprednisolone). Neuroleptics were not prescribed during this admission, so the only possible impact of medication would have been the chronic use of neuroleptics. Carbidopa/levodopa was added three weeks after transplantation; within two weeks, her improvement was remarkable. Normalization of her muscle tone, pull test, postural reflexes, gait, and cognitive status was observed. The psychomotor agitation evolved towards a picture of bradykinesia and postural tremor involving the upper limbs, head, and tongue. The transplantation is certainly the leading factor accounting for her clinical improvement; manganese-induced parkinsonism is renown to be refractory to levodopa administration, regardless of the posology or dosing. This hallmark of manganese-induced parkinsonism correlates with the nigrostriatal pathway preservation. We opted for a short trial of levodopa/carbidopa, monitoring closely the possibility of psychotic exacerbation [3].

She remained amnesic of the entire pretransplantation period, since the behavioural alteration. A follow-up brain MRI demonstrated a complete resolution of the pallidal hyperintensities. Carbidopa/levodopa was discontinued five months postoperatively, and the extrapyramidal features never recurred. A thorough neuropsychologic evaluation revealed that she performed at her baseline; she eventually returned to work and was eligible to drive.

Although manganese levels were not measured during the symptomatic period, manganese accumulation might have caused the symptomatology. In a state of chronic hepatopathy, hypoalbuminemia favours the passage of manganese through the blood-brain barrier. Manganese can stimulate the release of dopamine from presynaptic storage sites. Chronic manganese overload stimulates monoamine oxidase activity, increasing dopaminergic degradation and accumulation of its metabolite, homovanillic acid. This metabolite has been found in excessive quantities at autopsy in patients deceased with chronic hepatopathies [4–6]. Dichotomic observations characterize the impact of manganese deposition on dopamine neurotransmission. Nonetheless, studies describing manganese serum level by spectroscopy demonstrate a positive correlation between the serum level and the pallidal index (comparison between cortex and pallidal intensity on MRI) [7–9]. Manganese accumulates selectively within the basal ganglia; a positive correlation was also established clinically with the severity of the extrapyramidal symptoms, linking serum manganese directly to dopaminergic depletion [5, 6]. Manganese acts as a blocker of the D2 postsynaptic dopaminergic receptors, sparing nigrostriatal neuronal integrity. Dedicated specific neuroimaging modalities corroborate this hypothesis [10]. Even more, in vitro manganese exposure was shown to impact dopamine by reducing its uptake as well as its subsequent amphetamine efflux [11].

Our research and literature review highlighted this etiology retrospectively. We unfortunately did not document a manganese serum level at the time of the initial presentation. Despite that, we investigated our patient thoroughly, considering an extensive differential diagnosis as described above, mentioning hepatic encephalopathy and delirium. Her initial presentation, evolution throughout the transplantation process, and outcome are retrospectively strongly compatible with manganese overload.

## 2. Conclusion

Finally, in the presence of chronic liver disease, pallidal MRI hyperintensities, and neuropsychiatric symptoms, the medical work-up should include a serum manganese level. This case aims to increase awareness about this condition, which is potentially reversible after transplantation. Serum manganese dosing is readily available and inexpensive, and it may reveal the etiology rapidly, preventing further unnecessary investigations. The prognosis is also reassuring in this context and would indicate the urgency to proceed with a liver transplantation. Even if common pathologies predominate, we can identify the exceptions with insight, judgement, and vigilance.

## References

[1] Burkhard P. R., Delavelle J., Du Pasquier R., Spahr L. (2003). Chronic Parkinsonism Associated With Cirrhosis. *JAMA Neurology*.

[2] Perri G. A. (2016). Complications of end-stage liver disease. *Can Fam Physician*.

[3] Herrero Hernandez E., Discalzi G., Valentini C. (2006). Follow-up of patients affected by manganese-induced Parkinsonism after treatment with CaNa2EDTA. *NeuroToxicology*.

[4] Butterworth R. F., Spahr L., Fontaine S., Layrargues G. P. (1995). Manganese toxicity, dopaminergic dysfunction and hepatic encephalopathy. *Metabolic Brain Disease*.

[5] Forton D. M., Patel N., Prince M. (2004). Fatigue and primary biliary cirrhosis: Association of globus pallidus magnetisation transfer ratio measurements with fatigue severity and blood manganese levels. *Gut*.

[6] Ikeda S., Yamaguchi Y., Sera Y. (2000). Manganese deposition in the globus pallidus in patients with biliary atresia. *Transplantation*.

[7] Shah N. J., Neeb H., Zaitsev M. (2003). Quantitative T1 Mapping of Hepatic Encephalopathy Using Magnetic Resonance Imaging. *Hepatology*.

[8] Spahr L., Butterworth R. F., Fontaine S., Bui L., Therrien G., Lebrun L. H. (1996). Increased blood manganese in cirrhotic patients: Relationship to pallidal magnetic resonance signal hyperintensity and neurological symptoms. *Hepatology*.

[9] Verhoeven W. M., Egger J. I., Kuijpers H. J. (2011). Manganese and acute paranoid psychosis: a case report. *Journal of Medical Case Reports*.

[10] Guilarte T. R. (2010). Manganese and Parkinson's disease: a critical review and new findings. *Environmental Health Perspectives*.

[11] Roth J. A., Li Z., Sridhar S., Khoshbouei H. (2013). The effect of manganese on dopamine toxicity and dopamine transporter (DAT) in control and DAT transfected HEK cells. *NeuroToxicology*.

